# Analysis of population-specific pharmacogenomic variants using next-generation sequencing data

**DOI:** 10.1038/s41598-017-08468-y

**Published:** 2017-09-04

**Authors:** Eunyong Ahn, Taesung Park

**Affiliations:** 1 Interdisciplinary Program of Bioinformatics, Seoul National University, Seoul, 151-747 Korea; 2 Department of Computer Science, Technion − Israel Institute of Technology, Haifa, 3200003 Israel; 3 Department of Statistics, Seoul National University, Seoul, 151-747 Korea

**Keywords:** Statistical methods, Genetics research

## Abstract

Functional rare variants in drug-related genes are believed to be highly differentiated between ethnic- or racial populations. However, knowledge of population differentiation (PD) of rare single-nucleotide variants (SNVs), remains widely lacking, with the highest fixation indices, (F_st_ values), from both rare and common variants annotated to specific genes, having only been marginally used to understand PD at the gene level. In this study, we suggest a new, gene-based PD method, PD of Rare and Common variants (PDRC), for analyzing rare variants, as inspired by Generalized Cochran-Mantel-Haenszel (GCMH) statistics, to identify highly population-differentiated drug response-related genes (“pharmacogenes”). Through simulation studies, we reveal that PDRC adequately summarizes rare and common variants, due to PD, over a specific gene. We also applied the proposed method to a real whole-exome sequencing dataset, consisting of 10,000 datasets, from the Type 2 Diabetes Genetic Exploration by Next-generation sequencing in multi-Ethnic Samples (T2D-GENES) initiative, and 3,000 datasets from the Genetics of Type 2 diabetes (Go-T2D) repository. Among the 48 genes annotated with Very Important Pharmacogenetic summaries (VIPgenes), in the PharmGKB database, our PD method successfully identified candidate genes with high PD, including *ACE*, *CYP2B6*, *DPYD*, *F5*, *MTHFR*, and *SCN5A*.

## Introduction

Rare variants with large effect sizes have been predicted to exist in the human genome^1, 2^; also the large effect sizes of these variants have actually been observed, using real data analysis, but without analysis of population differentiation (PD)^3, 4^. These rare variants tend to be evolutionarily recent alterations, having many functional variants, within drug-related genes (“pharmacogenes”), thought to be highly differentiated between populations^5, 6^. However, PD approaches, for identifying rare variants, remain quite lacking; to date, the highest frequency indices (i.e., Fst values), from both rare and common variants annotated to specific genes, were used only to nominally understand PD at the gene level^7, 8^. Considering the fact that F_st_ values are proportional to minor allele frequencies (MAFs), this gene-level F_st_ summary is mostly governed by effect sizes (here, the effect sizes are PD) of common variants. Moreover, since previous various established methods (e.g., XP-EHH, and iHS) mainly focus on identifying haplotypes, however, adjacent common variants can strongly affect the results, and thus severely limit the identification of loci in alleles with intermediate frequency^9, 10^. Furthermore, in those methods, many rare variants in datasets significantly affect their performance by increasing the number of switch errors in the phased haplotypes^11–13^. Also the method recently proposed by Berg and *Coop* ^14^ was mainly for detecting correlations between genetic values and specific environmental variables. As results, these methods are inappropriate for our primary objective, i.e., finding genes with a high level of PD resulting from natural selection, in very recent evolutionary history (based on sequenced data with a large number of rare variants). On the other hand, our proposed method, PD of Rare and Common variants (PDRC), captures PD of both rare and common variants, and summarizes the results at the gene level. In our method, even while a linkage disequilibrium block is not analyzed, we summarize the information in a functional block, and simultaneously focus on very recently selected rare variants in sequenced data, shedding light on inconsistently inherited traits.

Allele frequency differences in pharmacogenes, especially between Africans, Europeans, and Asians, explain the danger of extrapolating therapeutic outcomes from one ethnic group to another^15, 16^. For instance, while a new drug may be approved by one specific nation’s health regulatory body, many other governments still require their own clinical studies, for their own citizens^17^. To that end, for ethnic PD differences, the PharmGKB database (www.pharmgkb.org) provides lists of Very Important Pharmacogene (VIPgene) summaries that associate with significant numbers of variant annotations and phenotypes (e.g., metabolism of, or response to, one or several drugs). Thus, although genome-wide variant mapping, to specific genes, was not a focus of our initial research, determining the PD of VIPgenes will enable investigators to find ethnic sensitivities, to therapeutic outcomes, for specific diseases. Additionally, PDRC can be extended to find very recent selection, resulting in considerable numbers of population-specific rare variants, and can even demonstrate associations between a gene and multiple phenotypes (“pleiotropy”), based on sequenced data. For instance, in the future, this approach will enable one to find tissue-specific or cancer-subtype-specific PD in somatic cells, based on data from the emerging technology of single-cell sequencing.

Since the advent of high-throughput (“next generation”) sequencing technology, it has been identified that, throughout the entire human genome, the majority of single-nucleotide variants (SNVs) are rare (86% of the total, with MAFs less than 0.5%)^18^, and population-specific (53–2%^18, 19^) (Supplementary Figure S1). Furthermore, rare variants are likely to exert important effects on pharmacogenetically driven phenotypes^20–22^. Evolutionarily, differences in gene expression, caused by rare variants, contribute to phenotypic diversity^23–25^. Thus, PD, throughout the human genome, can cause differential ethnic sensitivities to drug responses, through variations that are likely causal for specific genes’ expression and consequently, pathological phenotypes^26^. Thus, PD considerations are also crucial to drug development, approval, and treatment [PMID: 25669658]; global drug development, or bridging studies, are also important for new drug approval; identification of population-specific rare variants is essential for better understanding of genomic effects on ethnic specific drug responses.

Examination of PD of pharmacogenes requires methods that capture either variant-level or gene-level PD. When the scope of PD is expanded to a gene from a variant, our approach can achieve the following three advantages for population genetics research: (1) overcoming small effect-sizes, which cannot be detected by current variant based identification methods; (2) discovering genes under very recent selection, which also cannot be detected by current selection analyses; and (3) suggestion of additional levels of genetic evidence to explain differences in inherited traits, among populations. In this study, we compare our PDRC method to previously used PD determination algorithms, validating its superior performance at determining PD of numerous SNVs, as related to real whole-exome sequencing (WES) of 10,000 datasets, from the Type 2 Diabetes Genetic Exploration by Next-generation sequencing in multi-Ethnic Samples (T2D-GENES) initiative, and 3,000 WES datasets from the Genetics of Type 2 diabetes (Go-T2D) repository.

Herein, we demonstrate that previous PD analysis methods, for common variants^7, 27, 28^, are not appropriate for analyzing PD of rare variants. We then suggest a new PD analysis method, for rare variants, that is flexible in that it can combine rare and common variants efficiently. Our proposed PD method, Rare and Common variants (PDRC), is based on the Generalized Cochran-Mantel-Haenszel (GCMH) test for conditional independence in three-way contingency tables^29, 30^. Recently, the CMH test was used for rare variants analysis^31^. However, it has not been used for developing gene-level statistics, but only for meta-analysis, to summarize the statistics from each study^32^

## Results

### The proportion of rare variants in VIP genes

**Figure 1:**
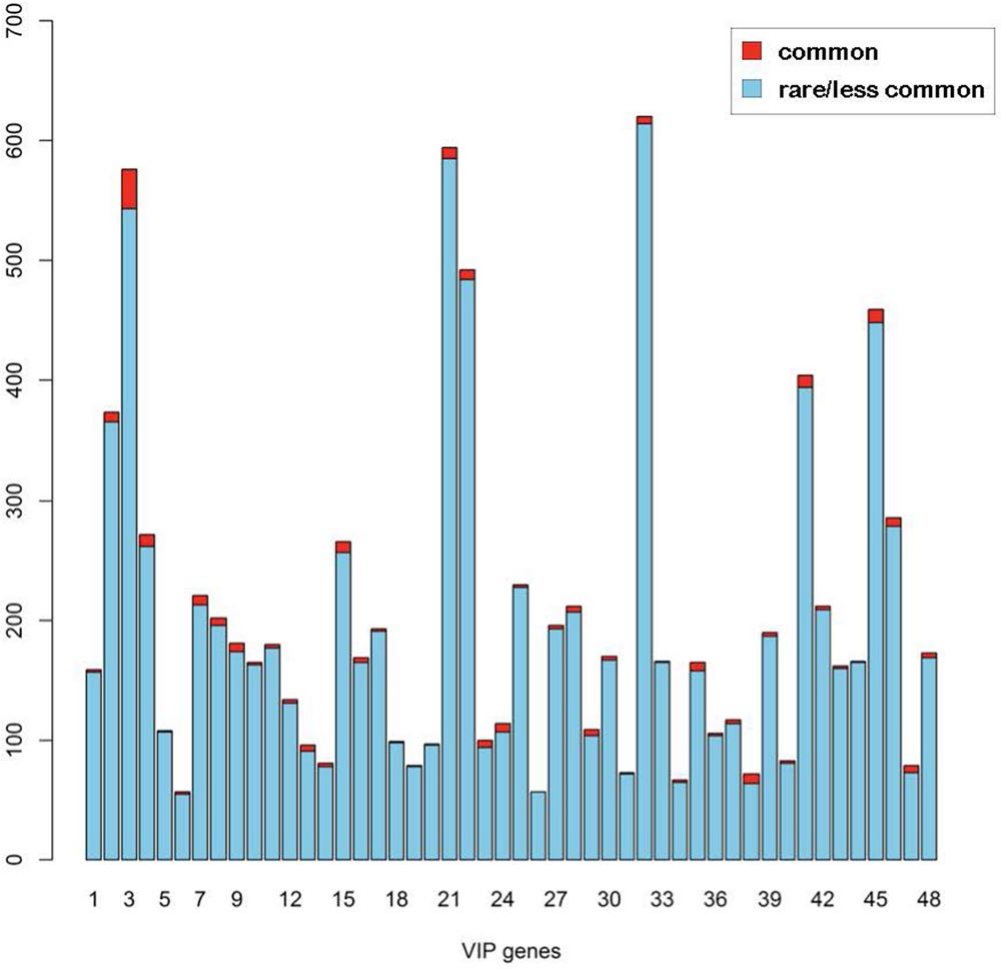
Rare variants in VIP genes. The blue-colored bars depict the number of rare/less common, and the bars are colored red to describe the amount of common variants, in the 48 VIP genes, of our datasets. Only a few of the variants in VIP genes in our datasets were common enough to be detected as highly differentiated variants, via F_st_.

We first procured whole exome sequencing (WES) datasets, the first consisting of 10,000 datasets, from the Type 2 Diabetes Genetic Exploration by Next-generation sequencing in multi-Ethnic Samples (T2D-GENES) initiative, and the second consisting of 3,000 datasets from the Genetics of Type 2 diabetes (Go-T2D) repository^33^. From the PharmGKB data analysis, we selected 48 genes, annotated as “Very Important Pharmacogenes” (VIPs) in PharmGKB **(PMID: 11908751)**. We removed two genes from the original 50 because one was on the sex chromosome and the other was not present in our WES data. Thus, we analyzed 48 VIP genes to determine those with high levels of population differentiation (PD). Since most of the variants in our WES data were less common or rare^34^, the selected 48 VIP genes mostly consisted of rare variants. The proportions of common variants in VIP genes are shown in Fig. 1. The overall proportion of common variants in VIP genes was 2.80% (max: 8.22%, min: 0%), and most MAFs of variants from the WES dataset were less than 0.05.

### Very small F_st_ from the variants in 48 VIP genes

We first calculated fixation index (F_st_) values for all the variants of each VIP gene. For this, we used Weir’s estimate, because it is unbiased, even when the sample sizes are unequal^35^. Since most of the variants were rare, their F_st_ values were also very small^36^. For this reason, the median F_st_ values for the 48 VIP genes were all less than 0.01, and only six VIP genes had at least one variant with an F_st_ greater than 0.25. These high F_st_ values were all from common variants, with MAFs greater than 0.05. For instance, rs1229984(T/C) showed the largest F_st_, 0.55, among the variants annotated to the 48 VIP genes, where the minor nucleotide for East Asian is T instead of C, and the frequency of the allele C of East Asian is 0.7445. The nine variants with F_st_ values greater than 0.25, from the six VIP genes, are summarized in Table 1. Although the MAFs of two variants, rs4846051 in *MTHFR* and rs6012687, in *PTGIS*, were not much higher than 0.05 (0.0532 and 0.0628, respectively), the F_st_ values of the two variants were 0.3000 and 0.2803, respectively. Moreover, racial or ethnic differences were found in the allele frequencies of rs4846051, related to the variation of response to methotrexate, in rheumatoid arthritis^37^.

**Table 1:** List of Variants with F_st_ values greater than 0.25.

Gene	SNP	MAF^a^	F_st_
*F5*	rs13306334	0.1656	0.4027
*F5*	rs9332658	0.1755	0.3763
*F5*	rs6020	0.2105	0.3365
*MTHFR*	rs4846051	0.0532	0.3000
*ADH1B*	rs1229984	0.1657	0.5591
*CYP3A4*	rs2687116	0.1303	0.4819
*CYP3A4*	rs2242480	0.2959	0.2803
*PTGIS*	rs6012687	0.0628	0.2581
*CYP2D6*	rs1081003	0.1346	0.3291

a Minor Allele Frequency of variants, as measured from our exome sequencing data.

**Figure 2:**
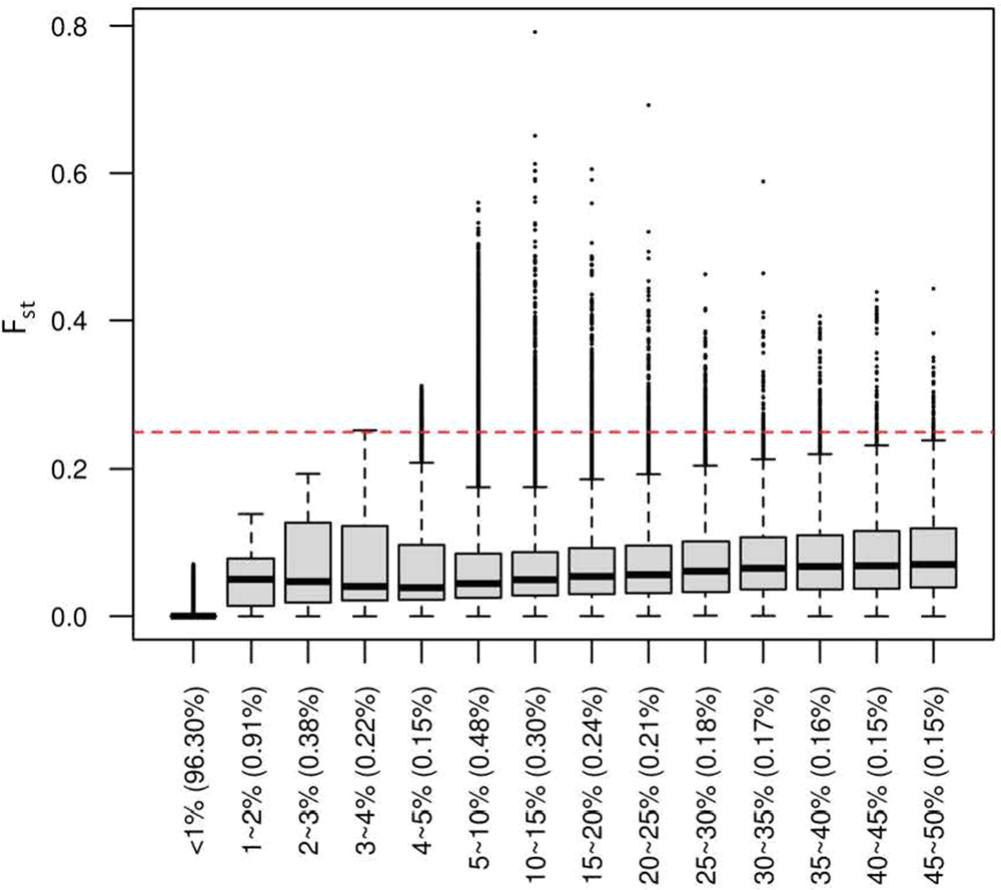
Boxplots of the F_st_ values of our WES data, according to MAFs of variants. The percentages of the variants which have the range of MAF over the number of all the variants in WES data are shown in x axis with parenthesis; the red line represents F_st_ cut-off 0.25 according to Wright’s F_st_ criteria^23^. Cardoso, *et al*.^23^ and Strauss, *et al*.^24^ defined a gene with PD if it contains at least one Single Nucleotide Polymorphism (SNP) with F_st_ value greater than 0.25.

Figure 2 shows that the distribution of F_st_ in our WES data varied according to the VIP genes’ MAFs. This also shows that the maximum F_st_, for all the variants with MAFs < 3%, was less than 0.25. Thus, F_st_ could not detect the variants of PD when their MAFs were smaller than 3%. In our WES data, 97.5% of the variants had MAFs smaller than 3%. Thus, the majority of variants in our data could not be identified, more specifically, as variants of PD. On the other hand, the common variants with MAFs >5% could be easily identified as variants of PD, by using F_st_.

For the VIP genes, 97.14% of variants had MAFs of less than 3%. Thus, the mere use of F_st_ made it difficult to identify VIP variants related to PD.

### SKAT analysis for all VIPgenes

We additionally performed the sequence kernel association test (SKAT)^38^ for all VIPgenes, even while our objective and the scope of analyses were limited to detecting differences between a pair of populations, at the gene-level. As expected, an association was found for most pairwise comparison of ancestral groups for VIP genes (472 from 480 pairs) after Bonferroni correction^39^. Specifically, for all VIPgenes, there were large differences in African Americans, due to their distinct genetic history^40^. Since SKAT can only perform for pairwise comparison of ancestral groups, these results are rather limited. For a more comprehensive comparison of all five ancestral groups, SKAT must be extended, to compare multiple groups.

### The PDRC tests for VIP genes

Three different weighting schemes, equal weights, inverse of MAF, and inverse of MAF^2^ (square of MAF) were adopted to compute PDRC test statistics. If we identified highly differentiated pharmacogenes among the 48 VIP genes by p-value, the number of those identified varied substantially in different weighting schemes. The *p*-values from PDRC tests, with equal weight, were very small, while the weight based on the inverse of MAF mitigated this phenomenon, as we already mentioned, such that the implementation of weight based on the inverse of MAF could reduce the false positive rate. Note that only two genes, *BRCA1* and *CYP2B6*, were identified by all nine of our different analyses (three strategies and three different weighting schemes), when we considered the p-values after Bonferroni correction. Considering that the equal weight is prone to increased false positive rates, the test result from the equal weighting scheme can be easily violated. Additionally, we evaluated the distribution of PDRC test statistics for each selection strategy and weighting scheme (Table 2), using all 18,281 genes in our WES datasets. We then calculated the 95^th^ percentile for all PDRC results. We selected the PD genes among the top 5% of genes and provided a more detailed description. The top 5% percentile of the PD measurement, from all the genes in the datasets, has often been used for PD detection^41–44^

**Table 2:** 95^th^ percentile of PDRC statistics for each selection strategy and weighting scheme.

Weighting Scheme	Selection strategy					
	All		Rare/Less Common		Protein Altering	
	Statistics	−log_10_(p-value)	Statistics	−log_10_(p-value)	Statistics	−log_10_(p-value)
Equal Weight	8667.888	>100	6689.85	>100	3791.395	>100
1/MAF	109.9717	22.44083	108.2756	22.07928	47.79004	9.300198
1/MAF^2^	54.35791	10.67047	54.17336	10.63187	28.91686	5.420125

Firstly, we obtained the gene-based summary statistics by using an ‘all variant’ selection strategy. Although the impact of synonymous variants on proteins was not confirmed, we assumed that all the SNPs possessed the potential for phenotypic variation. With the weights based on the inverse of MAF, PD values of all 48 genes were identified (*p*-value [Bonferroni, n = 48] < 0.05), making it possible to claim that all the VIP genes are highly differentiated (Table 3). On the other hand, considering the 95th percentile of the statistics, 6 genes, *ACE*, *CYP2B6*, *DPYD*, *F5*, *MTHFR*, and *SCN5A*, seemed to be specifically differentiated among the VIP genes. With weights based on the inverse of MAF^2^, PD values for all 48 genes identified (*p*-value [Bonferroni, n = 48] < 0.05), and the statistics from four genes, *CYP2B6*, *DPYD*, *F5*, and *SCN5A*, were larger than the 95^th^ percentile.

**Table 3:** 10 genes from PDRC tests, using ‘all’ or ‘rare/less-common’ variant selection strategies.

Gene	*PDRC statistics* (all)			*PDRC statistics* (rare/less common)			No. of variant		F_st_
	Weight:1	Weight: 1/MAF	Weight:1/MAF^2^	Weight:1	Weight:1/MAF	Weight:1/MAF^2^	Common	Rare	Max.
*DPYD*	685.41	**129.22**	**62.52**	1660.63	**131.13**	**62.52**	8	365	0.0731
*F5*	5561.83	**148.38**	**83.68**	5766.33	**149.95**	**83.68**	33	543	**0.4027**
*MTHFR*	1752.40	**117.12**	52.88	2814.08	**114.40**	52.88	10	262	**0.3000**
*SCN5A*	5608.12	**148.77**	**65.16**	**14227.78**	**149.18**	**65.16**	6	614	0.2084
CYP3A4	**13393.42**	13.15	7.88	304.07	12.15	7.88	2	160	**0.4819**
CYP3A5	**8674.38**	51.27	15.36	5369.90	50.29	15.36	1	165	0.2125
*EGFR*	40.72	107.68	53.18	**6729.38**	108.06	53.18	11	448	0.1594
*CYP2E1*	**18902.68**	68.42	24.36	**9627.48**	65.97	24.36	3	177	0.2128
*ACE*	3382.44	**154.36**	53.90	**16909.20**	**154.33**	53.90	9	585	0.1887
*CYP2B6*	96.18	**204.00**	**66.52**	1358.99	**204.16**	**66.52**	2	228	0.0728

Data are PDRC statistics yielded from the ‘all’ and ‘rare/less common’ strategies. Numbers of common and rare variants in each selected gene are described. Max. F_st_ is the maximal F_st_ value estimated from variants of each gene.

Secondly, when the PDRC test uses a ‘rare/less-common’ variant selection strategy, the results are similar to tests using all the variants (Table 3). Due to the fact that the weights, based on the MAFs, enable the PDRC test to put more weight on rare variants, PDRC tests using two different variant selection strategies, ‘all’ and ‘rare/less-common’ yielded the same list of genes.

Thirdly, when the PDRC test uses a ‘protein-altering’ variant selection strategy, a different list of genes was obtained (Table 4). In this case, the PDRC test identified PD in 10 and 12 genes with PD, with weights of the inverse of MAF and the inverse of MAF^2^, respectively (*p*-value [Bonferroni, n = 48] < 0.05). By the evaluated 95th percentile, one (*CYP2E1*) and six genes (*BRCA1*, *CYP2B6*, *DPYD*, *F5*, *MTHFR*, and *EGFR*) were selected with weights of the inverse of MAF and inverse of MAF^2^, respectively. Since the PDRC test combined all the effects of common and rare variants, while up-weighting rare variants, it successfully selected highly differentiated VIP genes that could not be detected by F_st_.

**Table 4:** Nine genes from PDRC tests, using ‘protein-altering’ variant selection strategy.

Gene Sym	*PDRC statistics* (protein-altering)			No. of variant			F_st_
	Weight:1	Weight:1/MAF	Weight:1/MAF^2^	Non Protein altering	Protein altering	Protein altering common	Max.
*DPYD*	724.50	5.32	**32.22**	229	144	3	0.0731
*F5*	2280.62	0.90	**43.78**	271	305	13	**0.4027**
*MTHFR*	958.49	4.22	**32.22**	181	91	3	**0.3000**
ADH1B	**8207.13**	7.33	2.38	78	39	1	**0.5591**
*EGFR*	970.60	17.55	**30.41**	323	136	1	0.1594
*CYP2E1*	2818.22	**51.69**	7.73	113	67	0	0.2128
*ACE*	**6183.58**	10.44	20.50	332	262	0	0.1887
*BRCA1*	1403.64	20.15	**29.45**	248	244	3	0.1377
*CYP2B6*	968.54	20.62	**31.96**	115	115	1	0.0728

Data are PDRC statistics yielded from the ‘protein altering’ strategy. Numbers of protein-altering and common & protein-altering variant in each selected gene are described. Max. Here, F_st_ is the maximal F_st_ value estimated from the variants of each gene.

### Genes identified via PDRC test with supporting evidence

From our PDRC test results using an ‘all’ or ‘rare/less-common’ variant selection strategy, PDs for all 48 VIP genes were identified, and supported by known PD values in many SNPs of VIP genes, based on microarray-derived previous research^45^. When we additionally evaluated the 95^th^ percentile of PDRC statistics, with weights based on either inverse of MAF or MAF^2^ (square of MAF) six genes, *BRCA1*, *CYP2E1*, *ACE*, *CYP2B6*, *SCN5A*, and *EGFR*, were selected, with no variants having F_st_ values greater than 0.25. Therefore, we propose these six genes to be specifically differentiated pharmacogenes, among other VIP genes. Besides, we found supporting evidence for high PD levels in these six genes, from either ethnic variation at the genetic^46–52^, or epigenetic, level^53–57^. The ethnic variations in genetic sequences of six genes have been reported, but the PDs of these genes could not be identified via F_st_. Genetic polymorphisms can be combined at the gene level, and their synergic variability could potentially affect the PD of either gene expression or phenotypes. For this reason, the gene-based statistic, PDRC, is advantageous for finding potential PDs at the epigenetic level. In fact, we specifically found evidence for PD at epigenetic level of three genes *CYP2B6*, *ACE*, and *SCN5A*; we will describe them in the following paragraph.

Hepatic *CYP2B6* expression is variable by ethnicity.^53^ Note that the maximum F_st_ among the SNPs in *CYP2B6* was less than 0.08, but the combined effect of the rare variants seemed to affect the variation in gene expression. In fact, there were only two common variants in *CYP2B6* from our WES data. This result shows that although a gene does not contain any SNPs, with large F_st_ values, it can be a gene with a high level of PD having large effects on expression levels. The Mantel-Haenszel odds ratios from each pair of ancestry groups are summarized in Table 5. Here, the European ancestry group is used as a baseline ancestry. African Americans tended to have more minor alleles than Europeans in our WES data (Mantel-Haenszel odds ratio, 2.33; 95% CI, 2.23 to 2.42). If we suppose that the minor allele potentially reduces a gene’s fitness, defined as the availability of a gene to perform a particular function, this may play a role in female African Americans having the lowest *CYP2B6* expression^53^.

**Table 5:** Mantel-Haenszel log odds ratios and confidence intervals of 3 genes.

*CYP2B6*	
	$\mathop{{\boldsymbol{\theta }}}\limits^{{\boldsymbol{\frown }}{}}$_*MHi*_ and 95% confidence interval
African American and European	2.3266 (2.2333, 2.4237)
East Asian and European	1.4618 (1.3844, 1.5434)
American Hispanic and European	1.2242 (1.1358, 1.3196)
South Asian and European	1.4167 (1.3406, 1.4972)
***ACE***	
African American and European	1.9047 (1.8543, 1.9562)
East Asian and European	1.5142 (1.4702, 1.5597)
American Hispanic and European	0.9461 (0.8972, 0.9977)
South Asian and European	1.6006 (1.5525, 1.6500)
***SCN5A***	
African American and European	2.9808 (2.8671, 3.0991)
East Asian and European	2.3238 (2.2309, 2.4203)
American Hispanic and European	1.3359 (1.2425, 1.4363)
South Asian and European	2.3298 (2.2336, 2.4302)

European are used as baseline to estimate the Mantel-Haenszel log odds ratios.

*ACE*, angiotensin-converting enzyme, yielded the second largest test statistic when ‘all’ variants were used, with the weight of inverse of MAF. The maximum F_st_ value among the SNPs in *ACE* was less than 0.2. *ACE* also was the predominant enzyme for bradykinin metabolism in human, where bradykinin is a potent endogenous, endothelium-dependent vasodilator. Consequently, reduced bradykinin expression could affect hypertension^58^. Since angiotensin is a vasoconstrictor, *ACE inhibitor*s are widely prescribed for the treatment of hypertension, although their efficacy has been reported to vary among different ethnic groups^48^. In addition, another previous study reported an interactive effect of ethnicity and an *ACE* gene insertion/deletion polymorphism associated with vascular reactivity^48^, and our current analysis result also showed ethnic sensitivity to ACE inhibitors. In our WES data, African Americans tended to have more minor alleles than Europeans (Mantel-Haenszel odds ratio = 1.90; 95% CI, 1.85 to 1.96), which is concordant with the phenotype of significantly attenuated vasodilation in Africans, when compared to Europeans^48^. The Mantel-Haenszel odds ratios and their confidence intervals are summarized in Table 5.

*SCN5A*, the sodium channel (voltage-gated) type V alpha subunit, includes variants with F_st_ values less than 0.14. However, the rate of cardiovascular disease (CVD), a *SCN5A*-related pathological phenotype, is likely related to ethnicity^49, 54, 55^. For instance, the prevalence of CVD is higher in rural southeastern regions of the US, with the largest African American population, compared to other regions^54, 55^. Similarly, Hispanics and African Americans have different genetic backgrounds, and patterns of linkage disequilibrium (LD), compared to populations of European descent^49–51^. Also, it is possible that genetic variation in *SCN5A* associates with electrocardiography (ECG), and cardiac traits that can vary, depending on the ancestral populations^55^, and this possibility is supported by the results of our analysis. African Americans, South Asians, and East Asians tended to have more minor alleles than Europeans in our WES data (Table 5). For instance, the Mantel-Haenszel odds of *SCN5A* from African Americans are 2.98 times higher than those from Europeans, at a 95% confidence level between 2.87 times higher and 3.10 times higher. This result also supports that PD of *SCN5A* potentially affecting ethnic variation of CVD prevalence, which is higher in African Americans than Europeans.

## Discussion

With the advent of low-cost, high-throughput sequencing technologies, a large number of rare variants have been identified in the human genome^34^, and it has been widely accepted that recent positive selection could result in between-group population differentiation (PD), in the human genome^59, 60^. Most rare variants are assumed to be driven by very recent positive selection, but have not yet reached fixation^60^, implying adaptation of modern-day humans to localized evolutionary pressure^60^. Similarly, when we identified pharmacogenes from whole-exome sequencing (WES) data, most rare variants were also population-specific. However, although most rare variants were identified as population-specific^61^, methods for measuring PD, using rare variant datasets, have not been well developed. In this study, we proposed a new PD analysis method, PD of Rare and Common variants (PDRC), based on the Generalized Cochran-Mantel-Haenszel (GCMH) test, for gene level analysis of PD in rare and common variants. Because PDRC can put more weight on rare variants, it enables us to avoid the minor allele frequency (MAF) dependency problem of chi-square statistics. Thus, PDRC can find significant genes, according to their PD, by placing more weight on rare variants. Since PD could potentially be used to identify distinct ethnic sensitivities in drug responses, we sought to find pharmacogenes, associated with PD, using our WES data. In addition to gene-level analysis, our PDRC method can be extended to find very recently selected genes. Such analyses will result in considerable identification of genes with population-specific rare variants, and even associations between genes and multiple phenotypes, based on sequencing data.

By evaluating measures for PD, we showed that both the chi-square test and F_st_ are dependent on MAF. For a given MAF, the maximum chi-square test statistic is shown to be proportional to MAF (S1 text^62^). The motivation of introducing the weight of MAF was to reduce the effect of MAF on the chi-square statistics. Without using the weight, the PDRC test statistic *L* ^2^ is also highly dependent on MAF^62^. However, by introducing weights, based on the inverse of MAF or MAF^2^, **B**_k_ (**n**_k_ −**μ**_k_) becomes less affected by MAF, as does the PDRC test statistic *L* ^2^. Similarly, this MAF-based weighting scheme has been widely used for genetic association tests that assign more weight to rare variants, and less weight to common variants^38, 63^. In our PD analysis, the introduction of weight tends to avoid reporting too many genes, with extremely small *p-*values, when the sample size is large.

Furthermore, if we assume that rare variants are driven by positive selection, the introduction of MAF weight is biologically meaningful, having only recently been introduced (but not yet fixed) in the genome. Under positive selection, MAF could be regarded as a rough measure of the age of the variant^38^. In this sense, the observable PD of rare and young variants is likely to be smaller than others, because the recent mutation could not have had sufficient time to be fixed in the population. Therefore, when we calculate a gene-level summary, it is biologically convincing to multiply a bigger weight to a more recently made variant.

In summary, we propose a new test (PDRC) for identifying genes having PD, based on next-generation sequencing (NGS) data. Our PDRC test provides a gene-based statistic for summarizing the effects of both rare and common variants. The possible impact of linkage- disequilibrium (LD), among rare variants in real WES datasets, on PDRC statistics, was investigated through permutation; it was also controlled by the implementation of weights, based on the inverse of MAF or MAF^﻿2﻿^. Also, through simulation studies, we demonstrated that PDRC tests well preserved type I error, which was not affected by the MAF distribution of genes, when the variants were considered independent of each other. Through an application to a real 13 K exome sequencing dataset, the PDRC test successfully identified pharmacogenes, with high levels of PD, from 48 Very Important Pharmacogenes (VIPs), according to different weighting schemes and selection strategies. To compare our results with known findings, at both the genetic and/or epigenetic levels, we specifically selected six genes, whose statistics were larger than the 95^th^ percentile, and simultaneously, without any variants, with F_st_ >0.25. Although the PD in these six genes could not be identified by F_st_ values, earlier studies have claimed the existence of PD at the genetic^46–52^, or epigenetic levels^53–57^, also supporting our findings^46–58, 64, 65^.

The gene-level-PD captured by our PDRC method can be used for the identification of recent adaptations by humans from sequencing data. For decades, genomewide research of natural selection has found that very recent beneficial genetic adaptation is often fixed in the human genome^66–68^. To that end, notable numbers of population-specific rare variants in our data also support recent adaptation that could provide selection of PD throughout the human genome.

Furthermore, If the ADME of a drug is closely related to a PD of pharmacogenes, we will be able to identify the scope of further investigation, and also devise ways researchers can screen drugs targeting genes with high PD, which can potentially be prone to be sensitive to ethnic factors, and also to suggest distinct pairs of ancestral allele groups, based on Mantel-Haenszel odds ratios^29, 69^. While it is hard to obtain whole genome sequencing data, at such large sample sizes, from the human genome, our method can be simply applied to WGS data. Longer computing time could be the only challenge to the application of our PDRC method to WGS data analysis. Besides, our method can be applied to other organisms, including viruses and bacteria, and more specifically the human microbiome. Specifically, RNA viruses tend to rapidly mutate, because of the lack of proofreading by their polymerase^70^. Therefore, when a target for vaccination is explored, our method potentially enables discovery of the most efficient ways to immunize the targeted host against the pathogen; and, considering that our PDRC method can capture the PD in bacterial genes, it could also be potentially applied to antibiotic resistance research.

In conclusion, our PDRC method precisely detects associations between multiple phenotypes and specific genes, based on sequencing data, and also facilitates interpretation of the possible biological impact, of rare variants, on specific traits of interest. This approach can accurately identify specific genes with high levels of PD, under very recent evolutionary selection. Here, we effectively identified highly population-differentiated pharmacogenes, by summarizing the effects of both rare and common variants, at the gene level. Such knowledge will greatly improve the design of therapeutic strategies for patients of distinct ethnicities, or even finding cancer-subtype-specific or tissue-specific somatic mutations, based on the emerging technology of single-cell sequencing. These results will also improve understanding of the recent evolution of SNVs, in specific genes, and possibly even indicate the selective pressure responsible for their respective associated phenotypes.

## Materials and Methods

### WES Datasets

We obtained whole exome sequencing (WES) data set from two consortia, T2D-GENES (Type 2 Diabetes Genetic Exploration by Next-generation sequencing in Ethnic Samples) and Go-T2D (the Genetics of Type 2 Diabetes Consortium)^33^. T2D-GENES is a NIDDK-funded research consortium that seeks to identify genetic variants for Type 2 Diabetes (T2D) through multiethnic sequencing studies. Go-T2D is a high-resolution study of type 2 diabetes genetic architecture through whole-genome sequencing of 2850 Europeans. T2D-GENES comprises three projects, from which we used data from Project 1. Project 1 seeks to assess whether less common variants play roles in T2D risk, in addition to similarities and differences in the distribution of T2D risk variants across ancestry groups. Presently, the T2D-GENES and Go-T2D initiatives are carrying out deep whole-exome sequencing (WES) of 13 K individuals, from which we used 12,844 unrelated individuals (6,474 cases, 6,370 controls) for our analysis. The total numbers of the five ancestry groups were as follows: 2025 African Americans, 2164 East Asians, 1938 American Hispanics, 2199 South Asians and 4518 Europeans. Among the five populations, cases and controls were well balanced for each ethnic group. From the European population, about 2000 samples were collected for case and control groups, respectively. Similarly, about 1000 samples were selected for case and control groups, respectively, from other ethnic groups. A more detailed description is given in the main paper^33^. Sequencing was completed at the Broad Institute using the Agilent (Santa Clara, CA) v2 capture reagent on HiSeq machines. After quality control, 3,130,381 variants matches to the datasets. In total, there were 62,489 common variants (MAF > 0.05) and 2,951,589 rare variants (0 < MAF < 0.01).

### PharmGKB Database

We used the PharmGKB (http://www.pharmgkb.org) database **(PMID: 11908751)** to select pharmacogenes for our PD study. This database is publicly available and encompasses clinical information, including dosing guidelines and drug labels, potentially clinically actionable gene-drug associations and genotype-phenotype relationships. Specifically, the very important pharmacogenes (VIP genes) represent the genes that greatly impact drug responses. These VIP genes have been widely used to decode the genomic effect on drug responses^71, 72^. VIP genes in PharmGKB were written by Scientific Curators, through extensive literature review, to provide a concise summary of key genes involved in drug responses, and whether these genes have been used for understanding pharmacogenomics^15^. Among 50 VIP genes in PharmGKB, one is on the sex chromosome and another was not included in our WES dataset. Thus, we used 48 genes for our analysis to identify pharmacogenes with PD.

### Test for Population differentiation for rare and common variants (PDRC)

For identifying genes with PD from the WES data, we proposed a new method, PDRC. The PDRC test is a gene-level summary test based on generalized Cochran-Mantel-Haenszel (GCMH) statistics^29, 30^. The main motivation of using the PDRC test was for extracting gene-based summary statistics that infer an average partial association between ancestral groups and genotypes. Initially, Cochran (1954) proposed a test, ‘average partial association,’ for a set of 2 × 2 tables, using a mean difference weighted across q tables, as determined by levels of the covariates^29, 30^. In particular, we considered detecting PD through the analysis of q s × r (s ≥ 2, r ≥ 2)) contingency tables under the multiple hypergeometric model assumption^30^. Here, q is the number of SNPs in a gene; s (=5) represents five ancestry groups, (1 for African Americans, 2 for East Asians, 3 for American Hispanics, 4 for South Asians and 5 for Europeans); r (=2) represents whether an allele is minor or major. For our analysis, we constructed a contingency table for each variant in a gene, and then combined them within a gene. Let ${\rm{k}}(=1,2,\cdots ,{\rm{q}})$ index a set of (${\rm{s}}\times {\rm{r}}$) contingency tables, which correspond to the number of SNPs in a gene. Let ${\rm{i}}(=1,2,\cdots ,5)\,\,$index five ancestry groups and j (=1, 2) index minor or major alleles, respectively. Let ${n}_{k}^{\text{'}}=({n}_{11k},\cdots ,{n}_{1rk},\cdots ,{n}_{s1k},\cdots ,{n}_{srk})$, where ${{\rm{n}}}_{ijk}$ denotes the number of subjects in the sample jointly classified as belonging to the *i* ^th^ ancestry group, the *j* ^th^ allele category and the *k* ^th^ SNP table. In addition, let ${N}_{i.k}$ denote the marginal total number of subjects in the *i* ^th^ ancestry group. In that case, ${N}_{j,k}$ is the marginal total number of subjects with the *j* ^th^ allele category, and ${N}_{\mathrm{..}k}\,\,$ is the overall marginal total sample size in the *k* ^th^ SNP table.

Using the GCMH test by Landis *et al*.^30^, we introduced the weight (i.e. the inverse of the MAF) into the PDRC test, for handling both rare and common variants, as follows:

1 $${H}_{0}:\,{\theta }_{i{j}_{1}(k)}={\theta }_{i{j}_{2}(k)}={\theta }_{i{j}_{3}(k)}={\theta }_{i{j}_{4}(k)}=1$$

2 $${n}_{k}={({n}_{11k},{n}_{12k},\ldots ,{n}_{1,j-1,k},\ldots ,{n}_{i-1,j-1,k})}^{\text{'}}$$

3 $${\mu }_{k}={({n}_{1.k}{n}_{.1k},{n}_{1.k}{n}_{.2k},\ldots ,{n}_{I-1,.,k}{n}_{.,J-1,k})}^{\text{'}}/{n}_{\mathrm{..}k}$$

4 $${\rm{cov}}({n}_{ijk},\,{n}_{i^{\prime} j^{\prime} k})=\frac{{n}_{i+k}({\delta }_{ii^{\prime} }{n}_{\mathrm{..}k}-{n}_{i^{\prime} +k}){n}_{.jk}({\delta }_{jj^{\prime} }{n}_{\mathrm{..}k}-{n}_{.j^{\prime} k})}{{n}_{\mathrm{..}k}^{2}({n}_{\mathrm{..}k}-1)}$$

with $${\delta }_{ab}=1\,{\rm{hen}}\,{\rm{a}}={\rm{b}}\,{\rm{and}}\,{\delta }_{ab}=0\,\mathrm{otherwise}\,.$$

5 $${L}^{2}={[\sum _{k}{B}_{k}({n}_{k}-{\mu }_{k})]}^{\text{'}}{[\sum _{k}{B}_{k}{V}_{k}{{B}_{k}}^{\text{'}}]}^{-1}[\,\sum _{k}{B}_{k}({n}_{k}-{\mu }_{k})]$$

6 $${B}_{k}={w}_{k}\times {I}_{n},$$

where ${W}_{k}$ is a weight. Then ${L}^{2}\sim {X}_{df=4}^{2}$ under the null hypothesis of conditional independence. Note that although rare variants often did not exhibit strong linkage disequilibium (LD)^73, 74^, but independence was also assessed via permutation (S2 text). From our permutation results, weights based on the inverse of MAF reduced the rate of false positives. Therefore, implementation of this type of weights is recommended for analyzing WES datasets like ours.

We reported the weighted Mantel-Haenszel odds ratio^69^, ${\hat{\theta }}_{M{H}_{i}}$, to show which ancestral alleles are distinctly different from European alleles (S3 text). We introduced the weight into the Mantel-Haenszel odds ratio and estimated variance^69, 75^, as follows, using the last cells as a baseline.

7 $${\hat{\theta }}_{M{H}_{i}}=\frac{{\sum }_{k}{w}_{k}({n}_{i1k}\,\cdot \,{n}_{52k})/({n}_{i.k}+{n}_{5.k})}{{\sum }_{k}{w}_{k}({n}_{i2k}\,\cdot \,{n}_{51k})/({n}_{i.k}+{n}_{5.k})}$$

where, i = 1, 2, 3 and 4. For the estimation of variance of ${\hat{\theta }}_{M{H}_{i}}$, we extended the methodology by Robins *et al*.^75^ Landis *et al*. showed that ${B}_{k}={u}_{k}\otimes {v}_{k}$, where ${u}_{k}$ is a vector of row scores and ${v}_{k}\,$is a vector of column scores^30^. When ${u}_{k}={I}_{(i-1)}$ and ${v}_{k}={I}_{(j-1)}$, *L* ^2^ is the generalized CMH statistic for two nominal variables. In the PDRC test, three types of weights are used: equal weight, inverse of MAF and inverse of MAF^2^. More details on these weights are given in the Discussion.

### Simulation

We conducted a simulation to analyze the type-1-error rate of PDRC test for the analysis of population differentiation (PD) in genes. The null distributions (i.e., no PD in the genome), were generated from several scenarios (S4 Text).

### VIP gene analysis through PDRC test

**Figure 3:**
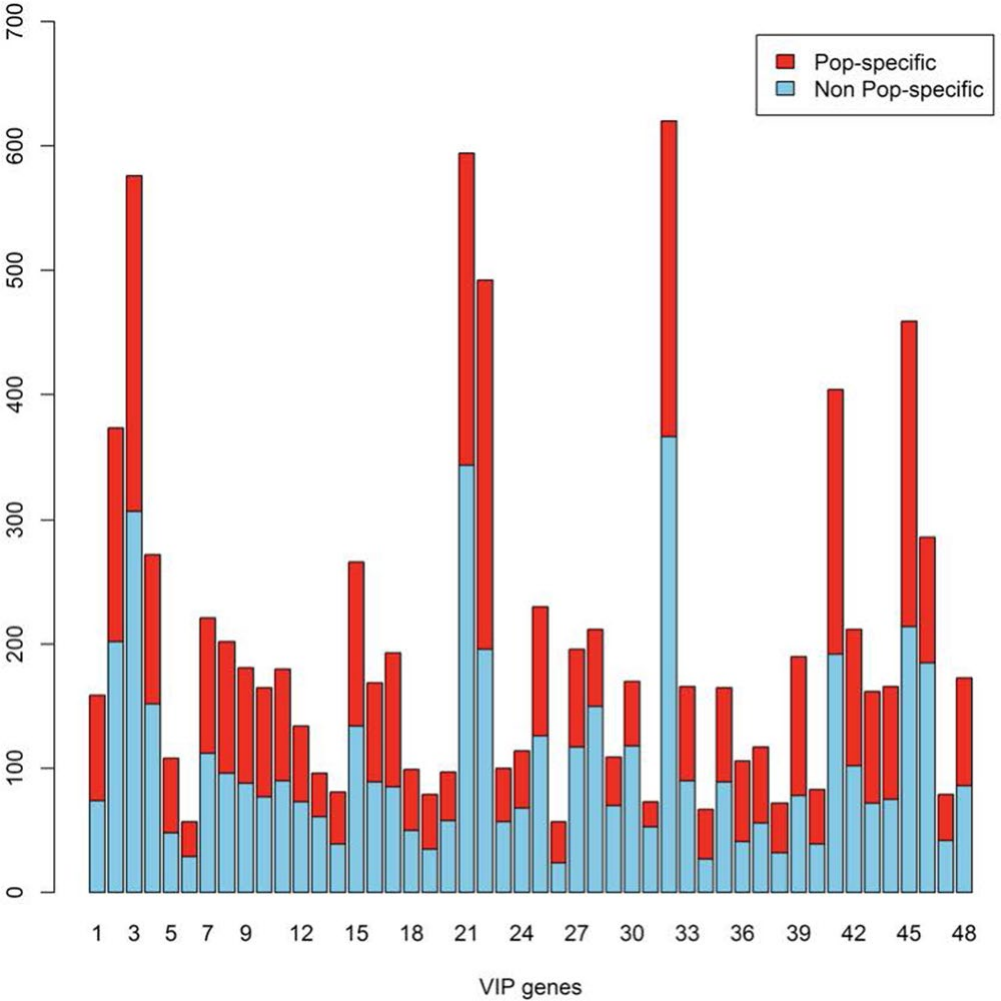
Population-specific variants in VIP genes. The bars are colored red to describe the numbers of population-specific variants, observed only from a single population, in our 48 identified VIP genes. Half of the variants in VIP genes in our datasets were population-specific rare variants.

According to the 1000 Genomes Project **(PMID: 21030618)**, 17% of low-frequency variants with MAF ranges of 0.5–5% were observed in a single ancestry group, and 53% of rare variants (MAF < 0.5%) were observed in a single ancestry group^19^ and similarly, half of the variants in VIP genes in our datasets were population-specific rare variants (Figs 2 and 3). Therefore, our studies of population-specific rare variants are also important for studying pharmacogenes with high PD. Therefore, we investigated the VIP genes in our WES data through the proposed PDRC test. Since the PDRC test statistics could summarize PD information from both rare and common variants, VIP gene analysis, via the PDRC test, has some flexibility in choosing variants in gene analysis, in order to identify genes with high PD.

### Variant selection strategy for specific genes

The process of selecting variants representing a specific gene is not straightforward. In our analysis, we considered the following three strategies for choosing variants: (1) all variants, including common and rare ones; (2) less common or rare variants; and (3) protein-altering variants. Since some of the variants do not alter the encoded protein, the phenotypic variation caused by genotypic variation might be summarized only by protein-altering variants^76^. However, non-protein-altering variants might also cause variation of gene expression, with phenotypic consequences^77, 78^. Lastly, less common and rare variants are expected to have larger effects than common variants^79^. Thus, these three strategies are used in the PDRC test for summarizing effects at the gene level.

## Electronic supplementary material

Supplementary information
