# Supplementary material for: Analysis of population-specific pharmacogenomic variants using next-generation sequencing data
Source: Sci Rep. 2017 Sep 4;7:8416. doi: 10.1038/s41598-017-08468-y (PMC5583360; doi:10.1038/s41598-017-08468-y)
Supplement: Supplementary file 1 — Supplementary information [file 41598_2017_8468_MOESM1_ESM.pdf]

Analysis of population specific pharmacogenomic variants using next generation sequencing data

Eunyong Ahn[1,2], Taesung Park[1,3*]

[1]Interdisciplinary Program of Bioinformatics, Seoul National University, Seoul, 151-747, Korea.

[2]Department of Computer Science, Technion − Israel Institute of Technology, Haifa, 3200003, Israel.

[3]Department of Statistics, Seoul National University, Seoul, 151-747, Korea.

[*]Corresponding Authors:

Taesung Park, Department of Statistics, Seoul National University, 1 Gwanak-Ro Gwanak-Gu, Seoul, 151-747, Korea, Tel: +82-2-880-8924; E-mail: tspark@stats.snu.ac.kr.

S1 Text. The dependence of F_{st} on MAF

Wu, *et al*. reported the PD among human genes using the HapMap data according to F_{st} [1]. They called the genes of PD if they contained at least one SNP with an F_{st} more than or equal to 0.6. However, it is not feasible that the F_{st} from rare variants exceed 0.6 and this method could not detect the population differentiation in rare variants. S2 figure shows the F_{st} in our WES data according to weighted average of MAF from each ancestry groups, and MAF from total population. The red line describes the maximum F_{st} when the MAF is given. The maximum F_{st} for MAF is defined when all the minor allele only exist in the ancestry group with the smallest sample size. If we assume there is no genotyping error, then we know how many loci are genotyped for each ancestry groups. In case of our data, the max F_{st} was identified when American Hispanics (1938) only have minor allele and other ancestry groups such as African Americans (2025), East Asians (2164), South Asians (2199), and Europeans (4518) do not have minor allele at all. The maximum F_{st} for the rare or less common variants (MAF < 0.05) is less than 0.36 and especially the maximum of F_{st} for the rare variants (MAF < 0.01) is less than 0.073. Therefore, for these variants, very high divergence ($0.25 < F_{st}$) or high divergence ($0.15 < F_{st} < 0.25$) in the wright's criteria need to be modified to find PD in rare variants.

The upper and lower bound of F_{st} when the MAF is given are already reported [2] and here we also showed that the maximum of F_{st} is bounded according to MAF when MAF is small enough through the equation. We used the initial definition of F_{st} which is developed by Wright for the simplification of proof. Since Wright developed this measure, many estimators has been proposed to estimate the F_{st} correctly under various situations. However, we only choose the Wright's F_{st} for our proof because other estimates are originated to estimate this parameter. Since Wright's F_{st} assume the ideal

situation with infinite allele and balanced sample sizes, this ideal condition would be different from the real world. However, we can assume the ideal condition theoretically, and our proof will be able to be extended to other estimators.

n_i denotes minor allele count of ancestry group i; N denotes the total genotyped allele counts; k denotes the total minor allele counts in population; m denotes the number of ancestry groups. Under Hardy Weinberg Equilibrium,

H_S = mean expected heterozygosity within random mating subpopulations = $2\overline{p_i q_i}$

H_T = expected heterozygosity in random mating total population = $2\bar{p}\bar{q}$

Wright's $F_{st} = \frac{H_T - H_s}{H_T}$

$$= 1 - \frac{H_T - H_s}{H_T}$$

$$= 1 - \frac{\frac{2}{m}\sum_{i=1}^{m}\left(\frac{n_i}{N/m}\left(1 - \frac{n_i}{N/m}\right)\right)}{2\left(\frac{k}{N}\right)\left(1 - \frac{k}{N}\right)}$$

$$= 1 - \frac{\sum_{i=1}^{m} m\left(n_i\left(\frac{N}{m} - n_i\right)\right)}{k(N-k)}$$

When k is less than N/m, $\exists n_i = k$ such that $i = 1 \cdots m$ and the minimum of nominator is $m\left(k\left(\frac{N}{m} - k\right)\right)$. Since $\sum_{i=1}^{m} n_i = k$, the minimum of nominator can be easily proved by Jensen's inequality [3]. Therefore, when k is less than N/m, the maximum of above equation is

$$= 1 - \frac{m\left(\frac{N}{m} - k\right)}{N - k}$$

$$= \frac{(\mathrm{m}-1)k}{N-k}$$
$$= \frac{(1-m)(\mathrm{N}-\mathrm{k}) + (m-1)\,\mathrm{N}}{N-k}$$
$$= (1-\mathrm{m}) + \frac{(m-1)N}{N-k}$$

As k is increasing; the denominator is decreasing; the maximum of F_{st} is increasing. Therefore, when we focus on the variants with small MAF, then k is less than N/m and the maximum of F_{st} is bounded according to their MAF

S2 Text. The permutation to confirm the distribution of PDRC

Considering small p-values from real data analysis, the distribution of PDRC may be claimed not to follow chi-square distribution. As an attempt to answer this issue, we permuted the ancestral allele information of each 48 VIP genes for 100 thousand times and calculated the PDRC statistics with three different weighting schemes. All the variants are used for this permutation regardless of their MAFs. In Supplementary S3 Fig, from several permuted data sets, the PDRC statistics without weight did not follow the chi-square distribution, but the PDRC statistics seem to be controlled by using weights as inverse of MAF, or inverse of MAF^2 (S4 Fig, and S5 Fig).

S3 Text. The variance of common odds ratio

$$\mathrm{var}(\log(\hat{\theta}_{MH_i})) = \frac{\sum_k w_k^{2}\left(n_{i1k}+n_{52k}\right)\left(n_{i1k}n_{52k}\right)/\left(n_{i.k}+n_{5.k}\right)^{2}}{2\left(\sum_k w_k\left(n_{i2k}\cdot n_{51k}\right)/\left(n_{i.k}+n_{5.k}\right)\right)^{2}}$$

$$+\frac{\sum_k w_k^{2}\left(\left(n_{i1k}+n_{52k}\right)\left(n_{i2k}n_{51k}\right)+\left(n_{i2k}+n_{51k}\right)\left(n_{i1k}n_{52k}\right)\right)/\left(n_{i.k}+n_{5.k}\right)^{2}}{2\left(\sum_k w_k\left(n_{i1k}\cdot n_{52k}\right)/\left(n_{i.k}+n_{5.k}\right)\right)\left(\sum_k w_k\left(n_{i2k}\cdot n_{51k}\right)/\left(n_{i.k}+n_{5.k}\right)\right)}$$

$$+\frac{\sum_k w_k^{2}\left(n_{i2k}+n_{51k}\right)\left(n_{i2k}n_{51k}\right)/\left(n_{i.k}+n_{5.k}\right)^{2}}{2\left(\sum_k w_k\left(n_{i2k}\cdot n_{51k}\right)/\left(n_{i.k}+n_{5.k}\right)\right)^{2}}$$

S4 Text. The simulation under the assumed null distribution

For the simulation to evaluate the type-1 error rate, we generated the data set without PD. We designed the data with four ancestral groups with 500 individuals and one ancestral group with 1000 individuals to assume a similar setting of sample sizes like our WES dataset. We also specified the number of SNPs in genes and assumed the distributions of MAF for a range of scenarios to investigate the potential effect of the number of variants in a gene and the distribution of MAF. In Scenarios 1 to 5, the rare or less common variants were generated; in Scenarios 6 to 10, the common variants were generated; in Scenarios 11 to 15, the variants were generated with the same MAF distribution as our WES data. The ancestral group information was randomly assigned to follow the null hypothesis, and 10^5 genes are simulated under fifteen different scenarios. For each setting of MAF distribution, 5 different numbers of SNP in a gene are assumed as following, 5, 10, 20, 50, and 100. Let $\mathrm{Gene}_{i,s}$ represent the *i*th Gene of scenario s for $i\in\{1, 2,\cdots, 99999, 100000\}$ and $SNP_{i,j,s}$ the *j*th SNP in $\mathrm{Gene}_{i,s}$ for

$j \in \{1, 2, \cdots, n_S\}$. The MAF of $SNP_{i,j,s}$, $p_{i,j,s}$, is sampled from $unif(0, 0.05)$ for scenarios 1 to 5, and from $unif(0.05, 0.5)$ for scenarios 6 to 10. For scenarios 11 to 15, $p_{i,j,s}$ is sampled from the MAF distribution of all 3,130,381 variants in our WES data sets (S1 Fig). Also, it is known that the p-values from the null distribution follow the uniform distribution and the distribution of simulated p-values can be investigated by QQ-plot. Supplementary Figures S6 to S8 show that the p-value of the PDRC test from the simulated data sets follows the uniform distribution when using three types of weights, 1, inverse of MAF, and inverse of MAF^2. According to these results, the type-1 error rate of PDRC tests seems to be reasonably controlled regardless the MAF values and the number of variants in a gene.

1 Wu, D. D. & Zhang, Y. P. Different level of population differentiation among human genes. *Bmc Evol Biol* **11**, 16, doi:10.1186/1471-2148-11-16 (2011).

2 Jakobsson, M., Edge, M. D. & Rosenberg, N. A. The relationship between F(ST) and the frequency of the most frequent allele. *Genetics* **193**, 515-528, doi:10.1534/genetics.112.144758 (2013).

3 Hölder, O. Ueber einen Mittelwertsatz. *Göttinger Nachr*, 38–47 (1889).

Legends to Supplementary Figures

S1 Fig. Histogram of $\log_{10}(MAF)$ from our WES data sets

S2 Fig. The maximum of F_{st} is bounded according to the MAF from total population

The red line represents theoretical maximum of F_{st}.

S3 Fig. QQ-plot results of PDRC without weight from the ancestral allele information permutation of VIP gene datasets

S4 Fig. QQ-plot results of PDRC with the weight as inverse of MAF from the ancestral allele information permutation of VIP gene datasets

S5 Fig. QQ-plot results of PDRC with the weight as inverse of MAF^2 from the ancestral allele information permutation of VIP gene datasets

S6 Fig. QQ-plot results from the simulation under null hypothesis 1

MAF of $SNP_{i,j,s}$, $p_{i,j,s}$, is sampled from $unif(0, 0.05)$ for scenarios 1 to 5

1: Number of SNPs in a Gene is 5. **2**: Number of SNPs in a Gene is 10. **3**: Number of SNPs in a Gene is 20. **4**: Number of SNPs in a Gene is 50. **5**: Number of SNPs in a Gene is 100. **A**: No weight, **B**: Weight is 1/MAF, **C**: Weight is $1/MAF^2$

S7 Fig. QQ-plot results from the simulation under null hypothesis 2

MAF of $SNP_{i,j,s}$, $p_{i,j,s}$, is sampled from $unif(0.05, 0.5)$ for scenarios 6 to 10

6: Number of SNPs in a Gene is 5. **7**: Number of SNPs in a Gene is 10. **8**: Number of SNPs in a Gene is 20. **9**: Number of SNPs in a Gene is 50. **10**: Number of SNPs in a Gene is 100. **A**: No weight, **B**: Weight is 1/MAF, **C**: Weight is $1/MAF^2$

S8 Fig. QQ-plot results from the simulation under null hypothesis 3

MAF of $SNP_{i,j,s}$, $p_{i,j,s}$, is sampled from the real MAF distribution of our WES data for scenarios 11 to 15

11: Number of SNPs in a Gene is 5. **12**: Number of SNPs in a Gene is 10. **13**: Number of SNPs in a Gene is 20. **14**: Number of SNPs in a Gene is 50. **15**: Number of SNPs in a Gene is 100. **A**: No weight, **B**: Weight is 1/MAF, **C**: Weight is 1/MAF^2

S1 Fig.

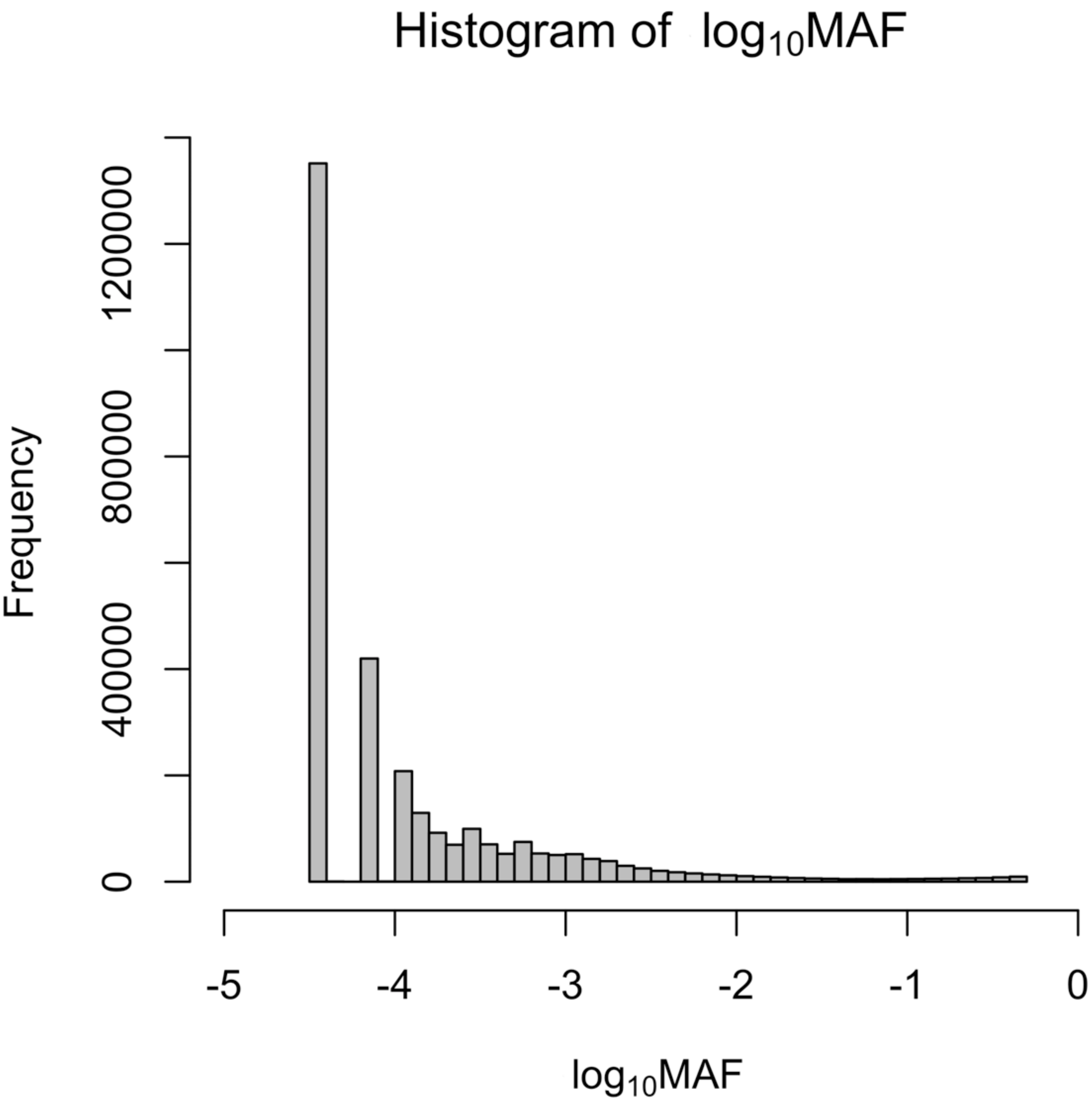

Histogram of $\log_{10}MAF$
Frequency
0
4000000
8000000
12000000
-5
-4
-3
-2
-1
0
$\log_{10}MAF$

S2 Fig.

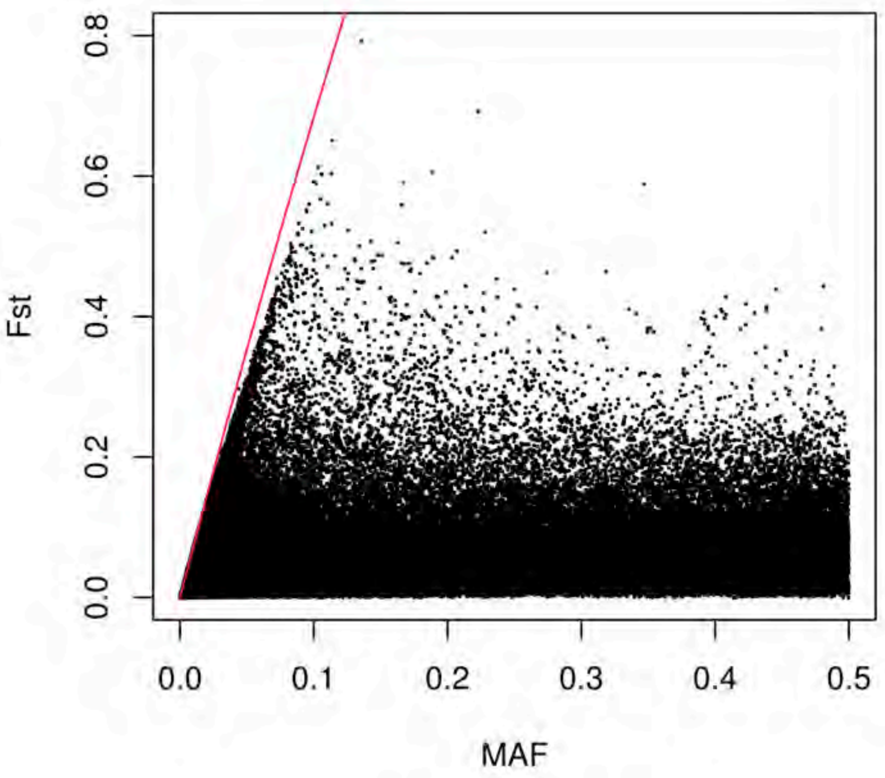

S3 Fig.

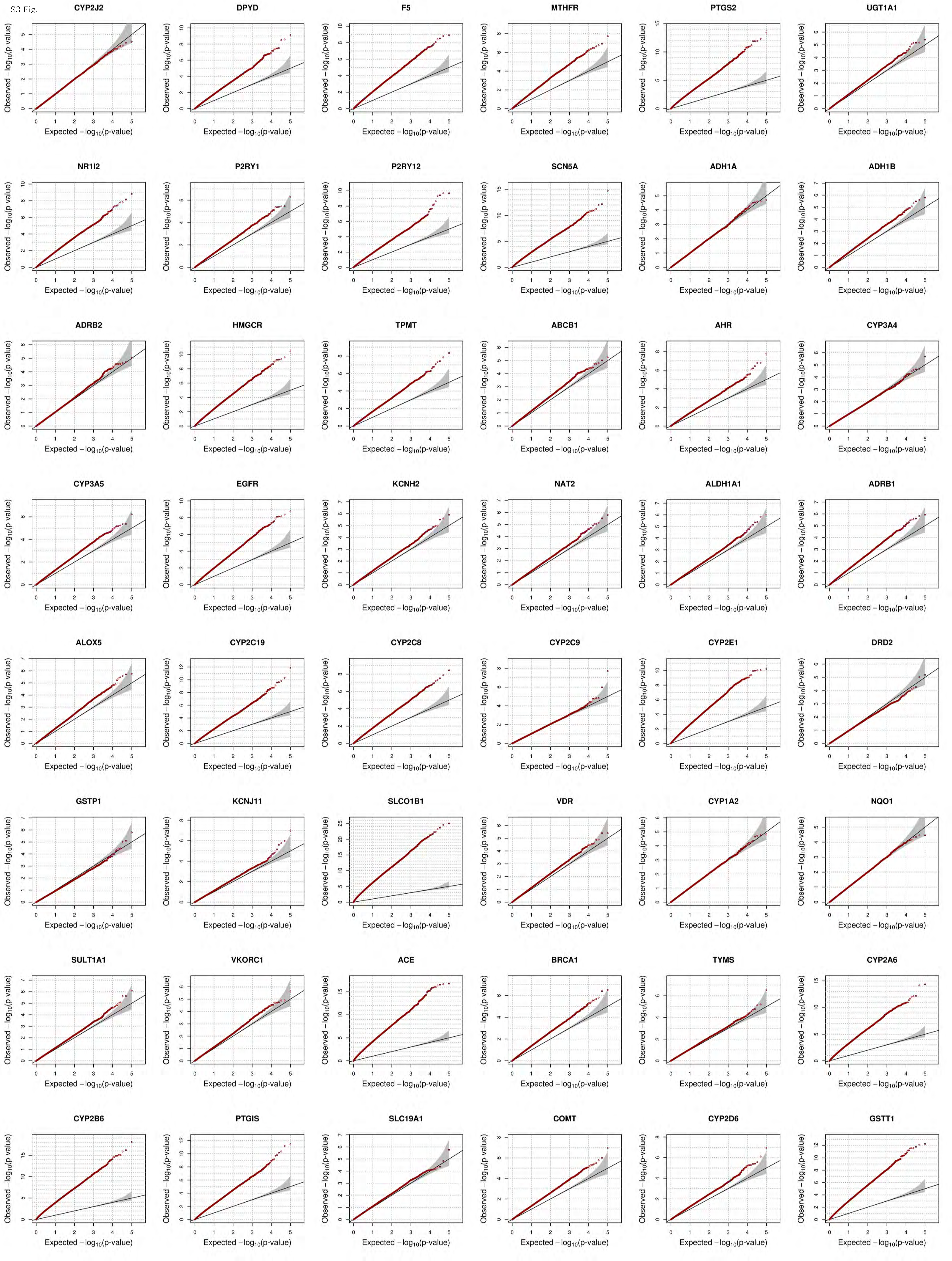
CYP2J2
DPYD
F5
MTHFR
PTGS2
UGT1A1
NR1I2
P2RY1
P2RY12
SCN5A
ADH1A
ADH1B
ADRB2
HMGCR
TPMT
ABCB1
AHR
CYP3A4
CYP3A5
EGFR
KCNH2
NAT2
ALDH1A1
ADRB1
ALOX5
CYP2C19
CYP2C8
CYP2C9
CYP2E1
DRD2
GSTP1
KCNJ11
SLCO1B1
VDR
CYP1A2
NQO1
SULT1A1
VKORC1
ACE
BRCA1
TYMS
CYP2A6
CYP2B6
PTGIS
SLC19A1
COMT
CYP2D6
GSTT1
Observed −log10(p-value)
Expected −log10(p-value)

S4 Fig.

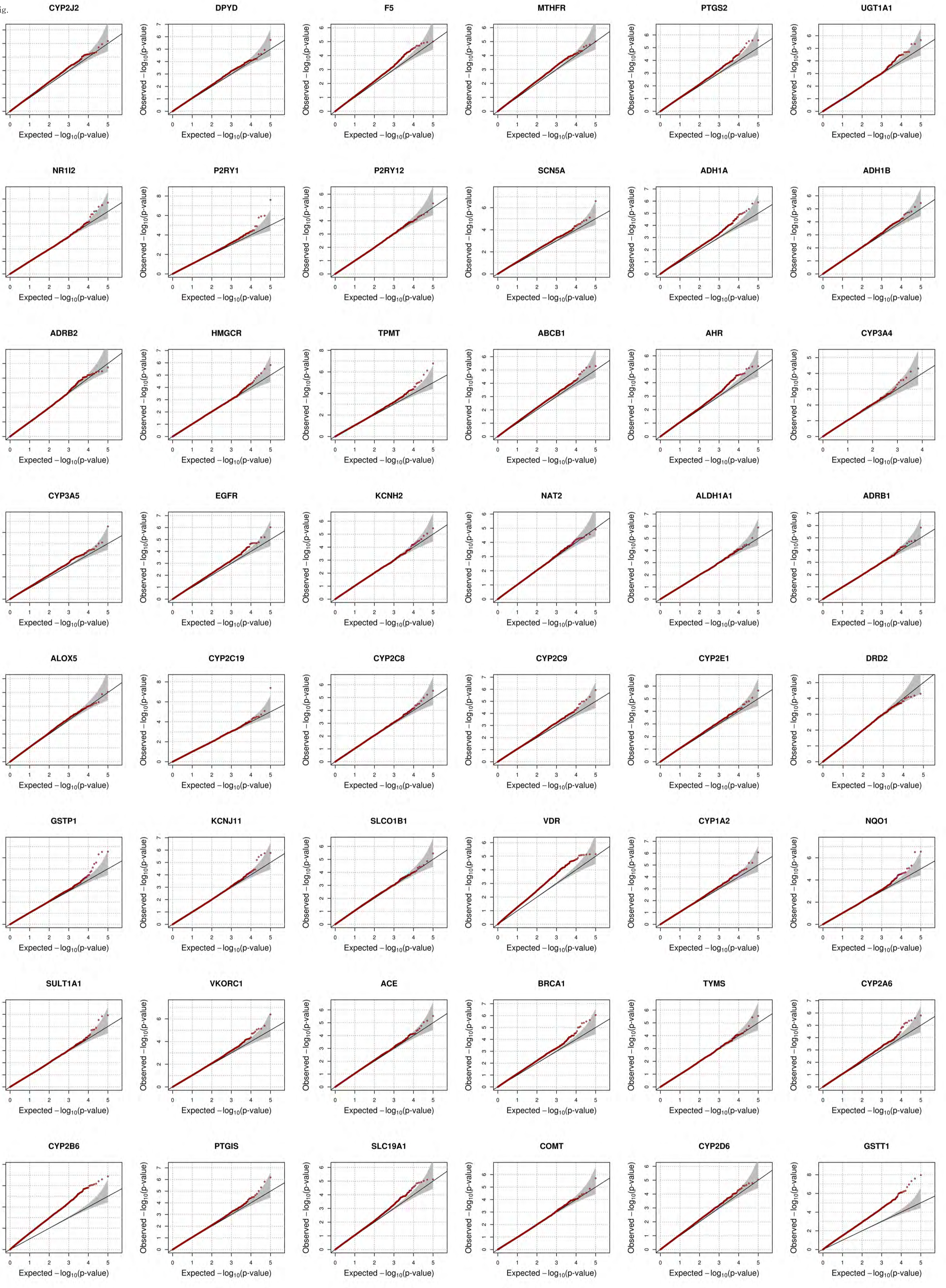
CYP2J2
DPYD
F5
MTHFR
PTGS2
UGT1A1
NR1I2
P2RY1
P2RY12
SCN5A
ADH1A
ADH1B
ADRB2
HMGCR
TPMT
ABCB1
AHR
CYP3A4
CYP3A5
EGFR
KCNH2
NAT2
ALDH1A1
ADRB1
ALOX5
CYP2C19
CYP2C8
CYP2C9
CYP2E1
DRD2
GSTP1
KCNJ11
SLCO1B1
VDR
CYP1A2
NQO1
SULT1A1
VKORC1
ACE
BRCA1
TYMS
CYP2A6
CYP2B6
PTGIS
SLC19A1
COMT
CYP2D6
GSTT1
Observed $-\log_{10}$(p-value)
Expected $-\log_{10}$(p-value)

S5 Fig.

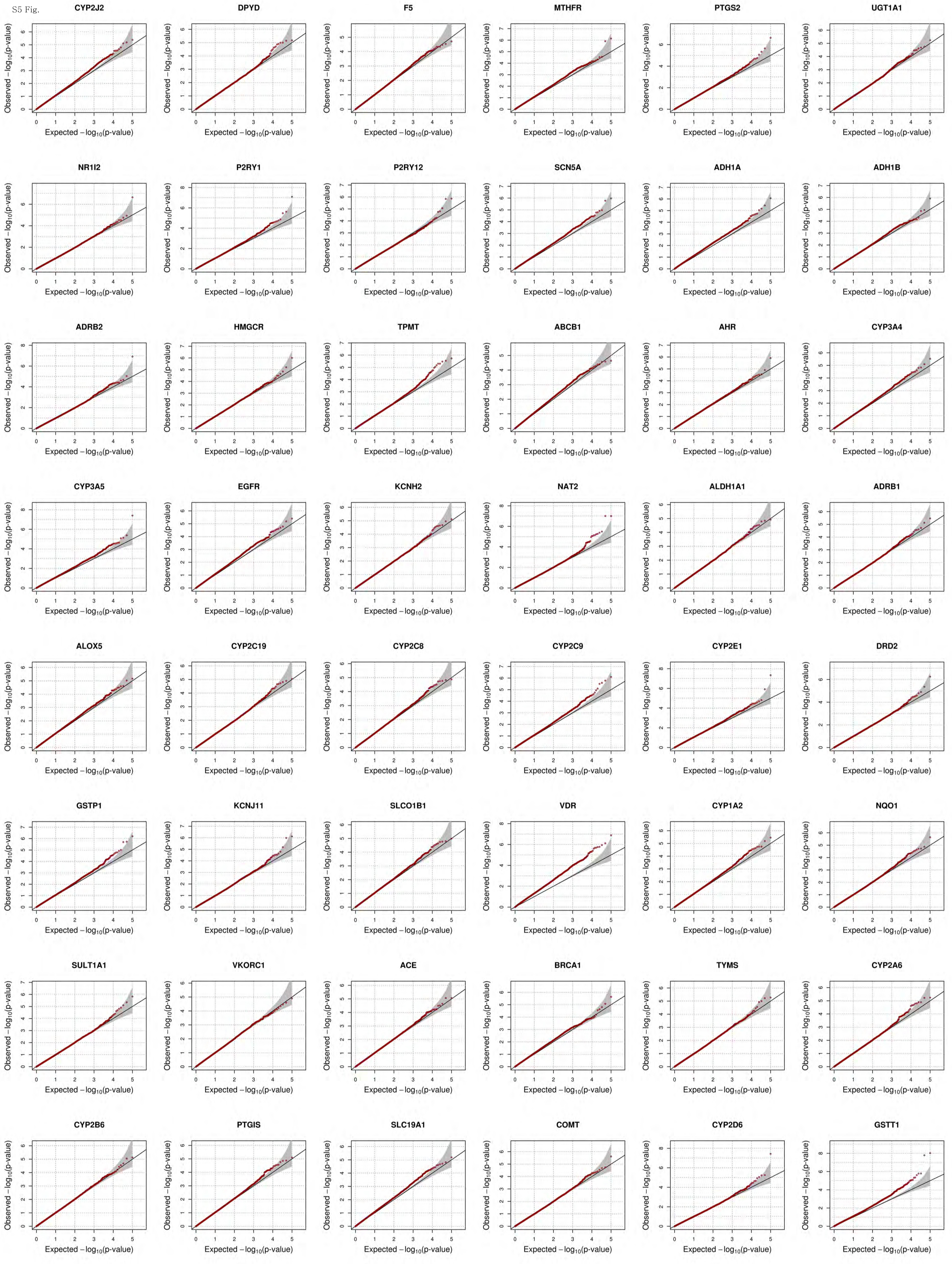
CYP2J2
DPYD
F5
MTHFR
PTGS2
UGT1A1
NR1I2
P2RY1
P2RY12
SCN5A
ADH1A
ADH1B
ADRB2
HMGCR
TPMT
ABCB1
AHR
CYP3A4
CYP3A5
EGFR
KCNH2
NAT2
ALDH1A1
ADRB1
ALOX5
CYP2C19
CYP2C8
CYP2C9
CYP2E1
DRD2
GSTP1
KCNJ11
SLCO1B1
VDR
CYP1A2
NQO1
SULT1A1
VKORC1
ACE
BRCA1
TYMS
CYP2A6
CYP2B6
PTGIS
SLC19A1
COMT
CYP2D6
GSTT1
Observed $-\log_{10}$(p-value)
Expected $-\log_{10}$(p-value)

S6 Fig.

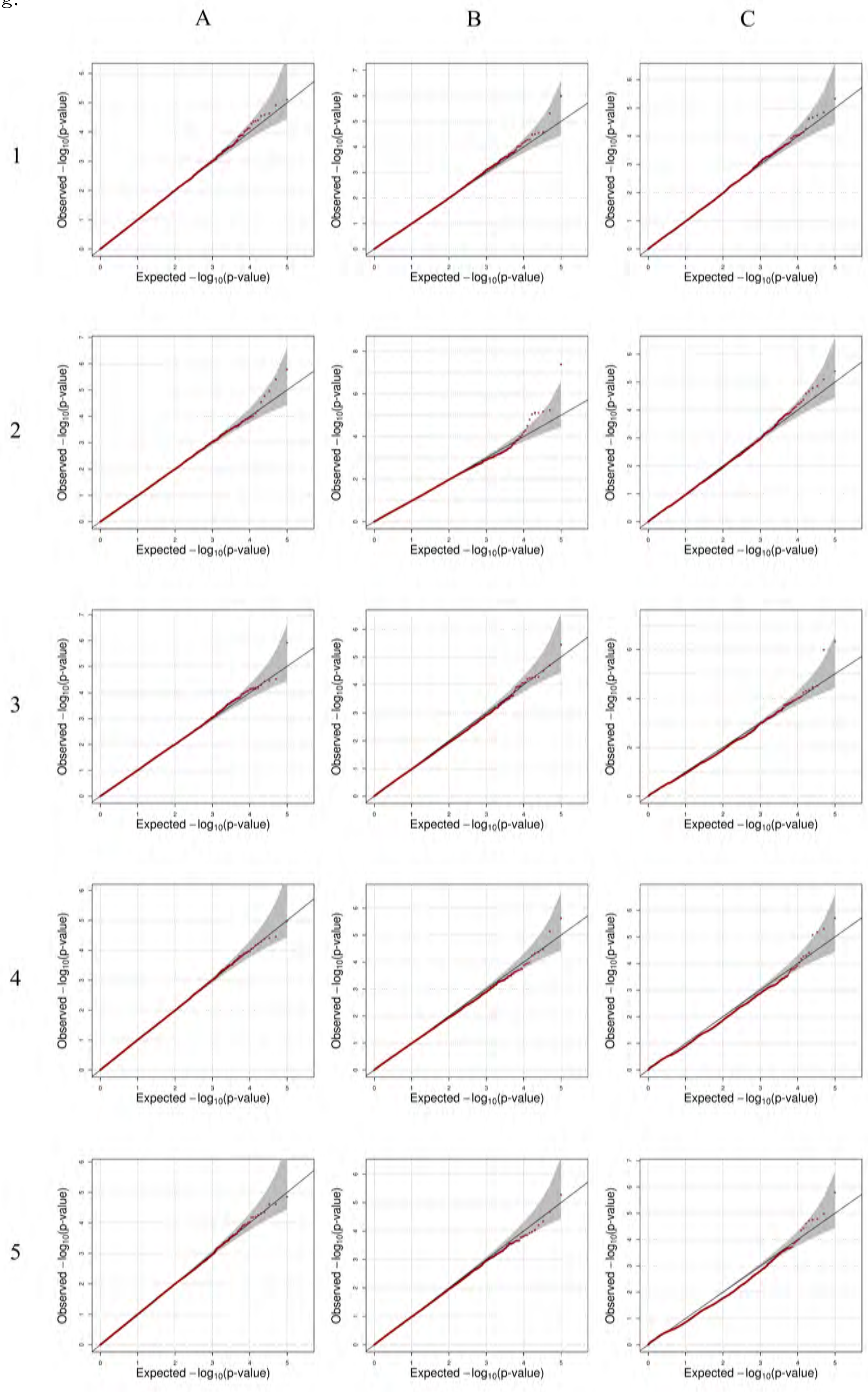

S7 Fig.

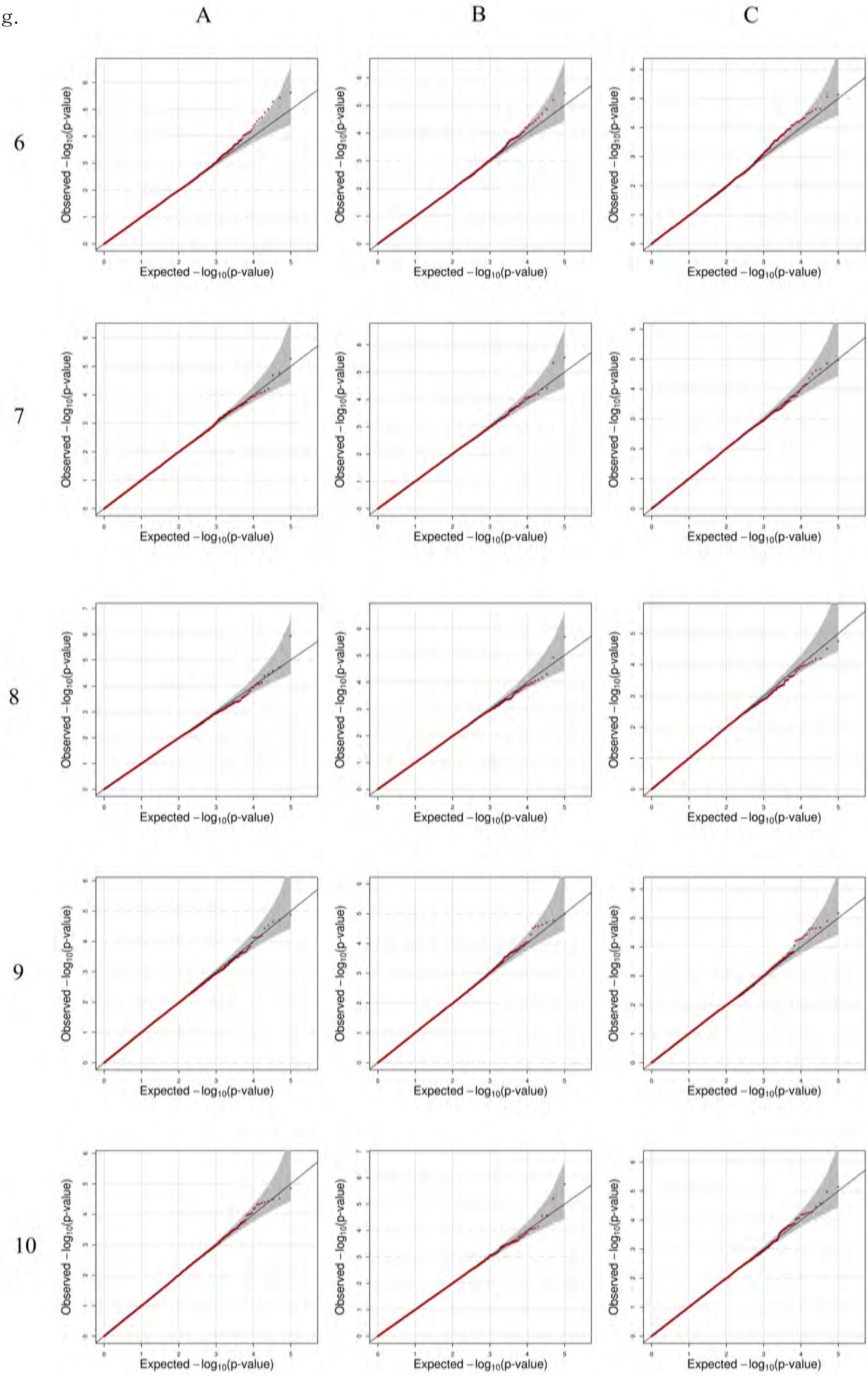

S8 Fig.

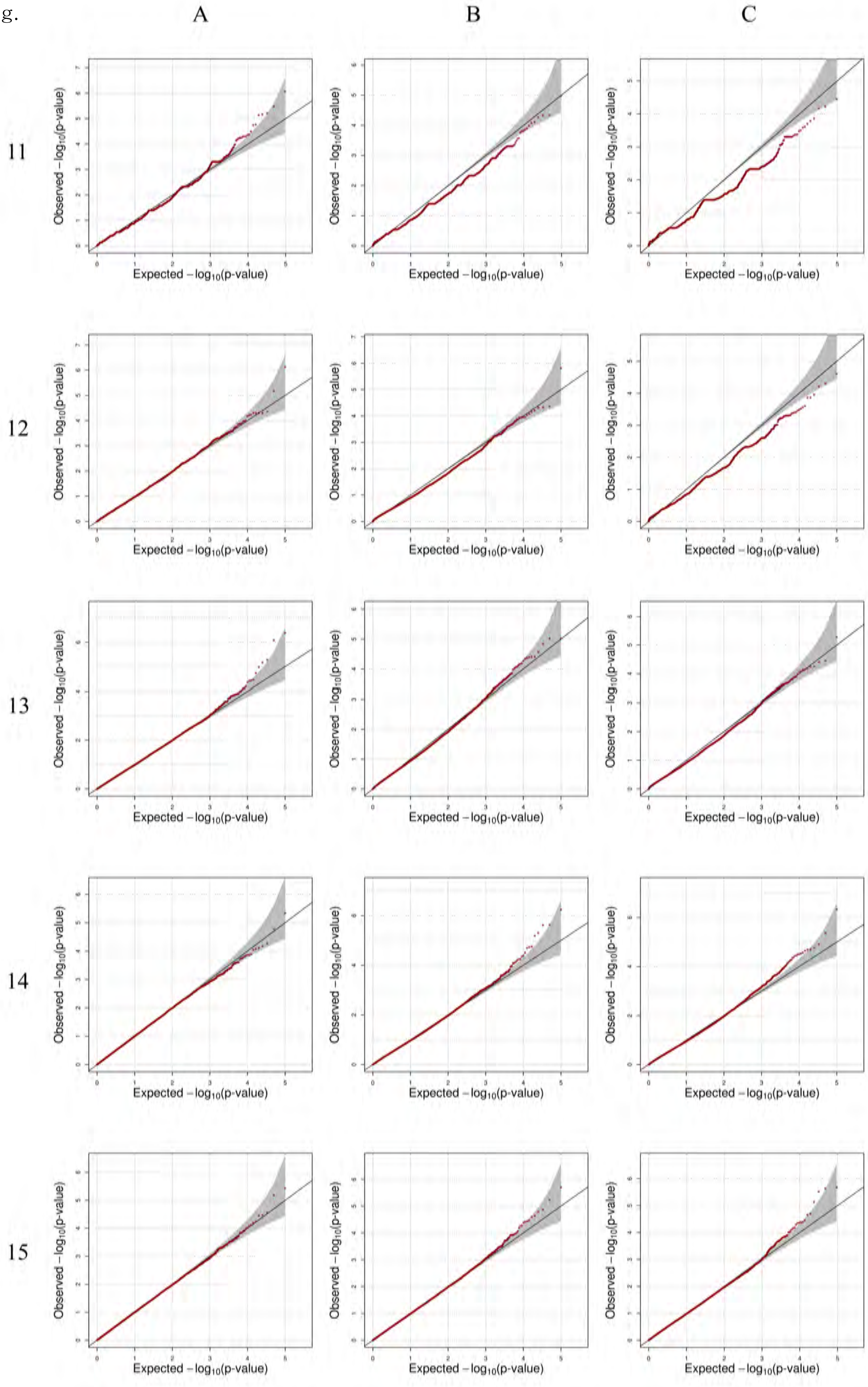
A
B
C
11
12
13
14
15
Observed −log10(p-value)
Expected −log10(p-value)
